# Surface electromyographic biofeedback versus neuromuscular electrical stimulation for post-stroke dysphagia: a systematic review and network meta-analysis

**DOI:** 10.3389/fneur.2025.1649961

**Published:** 2025-10-16

**Authors:** Na Li, Qianqian Jin, Yi Zhang, Li Zhang, Xiapei Peng

**Affiliations:** Department of Neurology, The Central Hospital of Wuhan, Tongji Medical College, Huazhong University of Science and Technology, Wuhan, China

**Keywords:** surface electromyographic biofeedback, neuromuscular electrical stimulation, stroke, dysphagia, network meta-analysis, systematic review

## Abstract

**Background:**

Post-stroke dysphagia (PSD) is a common complication with a high incidence rate, significantly impairing patients’ quality of life and health. Although conventional swallowing training is widely used, its efficacy depends on interindividual heterogeneity. Surface electromyographic biofeedback (sEMG-BF) is an emerging rehabilitation technology that shows promising potential in improving swallowing function. However, there is a lack of systematic and comprehensive evaluation and high-quality evidence to support its clinical application.

**Objective:**

This study aims to systematically evaluate and conduct a network meta-analysis comparing the efficacy of sEMG-BF, NMES, and conventional therapy in improving electrophysiological outcomes, swallowing function, and quality of life for patients with PSD.

**Methods:**

A systematic review and network meta-analysis were conducted by searching databases, including PubMed, EMBASE, Cochrane Library, Web of Science, and Scopus for prospective randomized controlled trials on the application of sEMG-BF in patients with PSD. We included randomized controlled trials that compared sEMG-BF, NMES, or conventional therapy in patients with PSD. The study focused on the effects of sEMG-BF on electrophysiological outcomes in these patients.

**Results:**

Six studies were included in the analysis. sEMG-BF was found to significantly increase mean amplitude (MD = 6.45, 95% CI: 3.53, 9.38) and reduce swallowing duration (MD = −0.22, 95% CI: −0.26, −0.18). The network meta-analysis revealed the following SUCRA ranking: sEMG-BF, neuromuscular electrical stimulation (NMES), and conventional therapy. sEMG-BF significantly improved the Standardized Swallowing Assessment (SSA) score (MD = −6.43, 95% CI: −9.74, −3.11). For Swallowing Quality of Life (SWAL-QOL), the pooled estimate was MD = 29.36 (95% CI: −14.96, 73.69), which did not reach statistical significance. The network meta-analysis demonstrated that sEMG-BF outperformed NMES and conventional therapy in improving swallowing function, consistent with direct comparison results.

**Conclusion:**

This study suggests that both sEMG-BF and NMES may provide benefits for PSD. Although sEMG-BF demonstrated superior effects in the majority of outcomes, the evidence is limited by small sample sizes and heterogeneity. Further high-quality trials are needed to confirm its efficacy. By enhancing the amplitude of electromyographic signals in swallowing-related muscles and improving muscle contraction capacity, sEMG-BF improves swallowing function; however, the pooled SWAL-QOL estimate was not statistically significant.

## 1 Introduction

Globally, stroke is the second leading cause of mortality and long-term impairment, representing a major public health challenge. It is frequently complicated by post-stroke dysphagia (PSD), with an estimated incidence of 34.4–80% (1–3). PSD not only significantly increases the risk of aspiration pneumonia but also elevates mortality (4). Dysphagia often leads to malnutrition and dehydration due to impaired swallowing, further exacerbating the deterioration of patients’ health (5). In addition, severe dysphagia restricts patients’ ability to participate in social activities, resulting in social isolation, diminished quality of life, and increased risk of depression (6, 7). Although conventional swallowing exercises, such as Shaker exercise and Mendelsohn maneuver, have been widely adopted, their efficacy is often limited by patients’ cognitive impairment or loss of proprioception, leading to significant interindividual heterogeneity in treatment outcomes (8, 9). Given these limitations, there is growing interest in exploring alternative therapeutic approaches.

In recent years, with advancements in neuroscience and rehabilitation medicine, various swallowing rehabilitation therapies have emerged. Surface electromyography biofeedback (sEMG-BF) therapy, a novel rehabilitation technique, has gained increasing attention and demonstrated promising potential in the treatment of PSD (10). sEMG-BF enhances the amplitude of electromyographic signals in swallowing-related muscles, improves muscle contraction capacity, and promotes upper esophageal sphincter opening and epiglottis elevation, thereby improving pharyngeal transit efficiency and bolus clearance while simultaneously enhancing airway protection mechanisms during deglutition (11–13). Furthermore, sEMG-BF can augment patients’ self-awareness and control over swallowing movements, promoting active participation in therapy and further optimizing treatment outcomes (14).

As a safe, effective, and feasible rehabilitation modality, sEMG-BF holds broad prospects for the treatment of PSD. However, systematic and comprehensive evaluation are still lacking, and high-quality evidence regarding its specific efficacy and advantages in this context remains limited. This study systematically evaluates the efficacy of sEMG-BF and NMES for PSD through network meta-analysis, comparing their therapeutic benefits against conventional therapy to establish evidence-based clinical recommendations.

## 2 Methods

### 2.1 Study design

Systematic review and network meta-analysis were conducted and reported in accordance with the PRISMA 2020 guidelines and the PRISMA extension for network meta-analyses (PRISMA-NMA). A dual methodological approach that combines systematic evidence synthesis with advanced network meta-analytical techniques was used to rigorously assess the therapeutic effectiveness of sEMG-BF for managing swallowing disorders following cerebrovascular accidents, aiming to provide high-quality evidence for clinical decision-making. This review was prospectively registered with the International Platform of Registered Systematic Review and Meta-analysis Protocols (INPLASY; Registration number: INPLASY202550028).

### 2.2 Literature search

A comprehensive literature search was conducted across five major databases, including Scopus, EMBASE, Web of Science, Cochrane CENTRAL, and PubMed/MEDLINE. The search encompassed all available records from each database’s inception through April 1, 2025, with language restrictions limited to English and Chinese. Search terms included “surface electromyography biofeedback,” “post-stroke dysphagia,” and related variations. Tailored search strategies were developed for each database to ensure comprehensiveness and accuracy.

For instance, the PubMed search strategy is as follows:

(“stroke” OR “cerebrovascular accident” OR “CVA” OR “brain attack”) AND (“dysphagia” OR “swallowing disorder” OR “swallowing difficulty” OR “feeding difficulty”) AND (“surface electromyography” OR “sEMG” OR “electromyography” OR “bioelectrical activity”).

### 2.3 Inclusion and exclusion criteria

The Population, Intervention, Comparison, Outcomes, Study Design (PICOS) framework was applied to determine eligible studies, as outlined in Table 1.

**Table 1:** PICOS criteria for literature inclusion.

Dimension	Detail
Population	Adults aged ≥18 years with post-stroke dysphagia
Intervention	Surface electromyography-guided swallowing facilitation (sEMG-SF)
Comparison	Conventional swallowing rehabilitation training or neuromuscular electrical stimulation (NMES)
Outcomes	Primary outcomes: Electromyographic signals of submental muscle groups (mean amplitude and swallowing duration); Secondary outcomes: Quality of life and swallowing function scores
Study design	Randomized controlled trials (RCTs)

#### 2.3.1 Exclusion criteria

Studies were excluded based on the following criteria: (1) non-randomized studies, including case reports and conference abstracts; (2) studies involving patients with comorbid neurodegenerative disorders (e.g., Parkinson’s disease) or head/neck tumors; (3) interventions incorporating invasive biofeedback techniques such as intraluminal manometry; (4) duplicate publications, studies with incomplete data, or those for which full texts were inaccessible; and (5) studies with insufficient sample sizes that did not allow for meaningful statistical analysis.

#### 2.3.2 Literature screening and data extraction

The study selection process was performed independently by two investigators using a dual-phase screening approach. In the primary phase, all retrieved citations underwent title and abstract review to identify potentially relevant publications, followed by comprehensive full-text assessment of selected articles in the secondary phase. Inter-rater disagreements were addressed through consensus discussions or arbitration by a senior researcher when required. A customized data collection template was used to capture key study elements, including publication metadata (author names, publication year, country of origin, and research design), population characteristics (sample size, demographic parameters, and stroke classification), intervention specifications (treatment frequency, duration, biofeedback parameters, and adjunct therapies), comparator details (sham procedures and standard care protocols), efficacy metrics (primary and secondary outcomes with corresponding assessment instruments), and temporal evaluation points (post-treatment follow-up intervals). This systematic approach ensured consistent and thorough data acquisition while minimizing selection bias. All extracted data were cross-verified by both researchers to ensure accuracy and completeness prior to analysis.

#### 2.3.3 Quality assessment

The methodological quality of the included randomized controlled trials was rigorously assessed using the Cochrane Collaboration’s revised Risk of Bias tool (ROB 2.0), which systematically evaluates potential biases across five critical domains. The tool examined the adequacy of random sequence generation and allocation concealment in the randomization process, potential biases introduced by deviations from the protocol, including non-adherence or unintended unblinding, handling of missing data with particular attention to dropout rates and adherence to intention-to-treat analysis, objectivity in outcome measurement through assessor blinding and instrument validity, and consistency between pre-specified and reported outcomes to detect selective reporting. Each trial received domain-specific judgments of “low risk,” “some concerns,” or “high risk” of bias, with these evaluations subsequently synthesized and presented through both summary tables and visual plots for comprehensive interpretation. This systematic approach enabled transparent evaluation of study quality while identifying potential limitations in the evidence base.

#### 2.3.4 Statistical analysis

The synthesis comprised two components: traditional pairwise meta-analysis and network meta-analysis (NMA).

#### 2.3.5 Traditional meta-analysis

For continuous outcomes (e.g., motor function scores), mean differences (MDs) with 95% confidence intervals (CIs) were calculated using sample sizes, means, and standard deviations. When studies reported medians and interquartile ranges, these values were converted to means and SDs using the method described by Wan et al. (15), with explicit notation of this transformation in the results. Heterogeneity was quantified via the *I*^2^ statistic and Cochran’s *Q* test; a random-effects model was employed if *I*^2^ exceeded 50%. Subgroup analyses explored potential sources of heterogeneity (e.g., stroke phase and intervention dosage).

#### 2.3.6 Network meta-analysis

A frequentist framework was adopted using STATA 17.0’s network package (StataCorp, College Station, TX, USA). The geometry of the intervention network was visualized with nodes (treatments) sized by sample volume and edges (direct comparisons) weighted by study count. Consistency between direct and indirect evidence was tested via node-splitting (*p* > 0.10 indicating agreement). Treatment rankings were derived from surface under the cumulative ranking curve (SUCRA) values, where higher percentages denoted superior efficacy.

## 3 Results

### 3.1 Literature screening process

**Figure 1:**
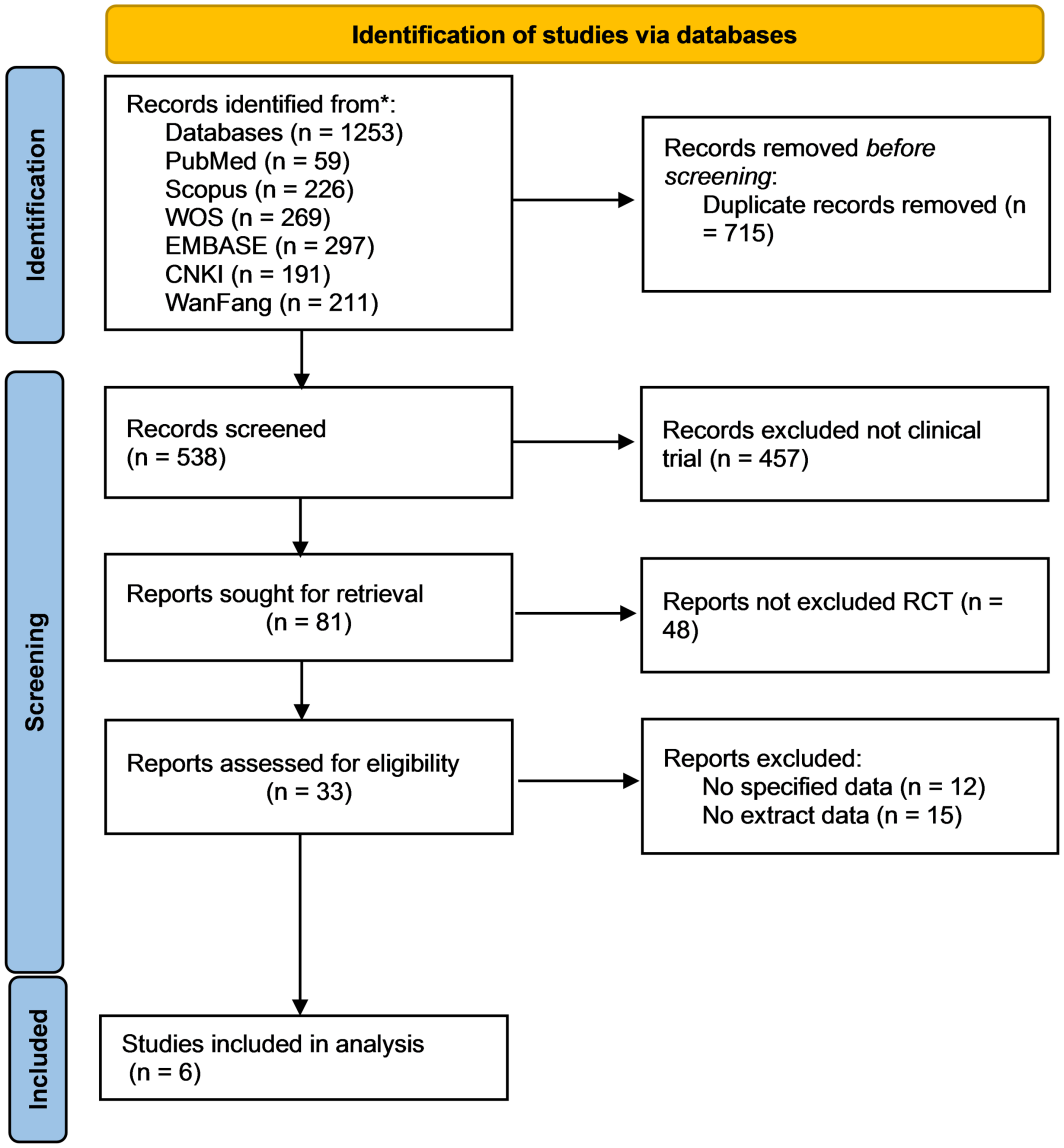
Literature screening process.

A systematic search was conducted across six databases (PubMed, Scopus, Web of Science, EMBASE, CNKI, and WanFang), yielding 1,253 records. After removing 715 duplicate records, 538 records underwent preliminary screening. Following the exclusion of 457 non-clinical studies, 81 full-text articles were selected for further evaluation. Subsequently, 48 non-randomized controlled trials were excluded, leaving 33 reports for full-text review. After detailed assessment, 27 studies were excluded due to insufficient data or inability to extract relevant outcomes, resulting in the final inclusion of six studies for meta-analysis (Figure 1).

### 3.2 Characteristics of included studies

**Figure 2:**
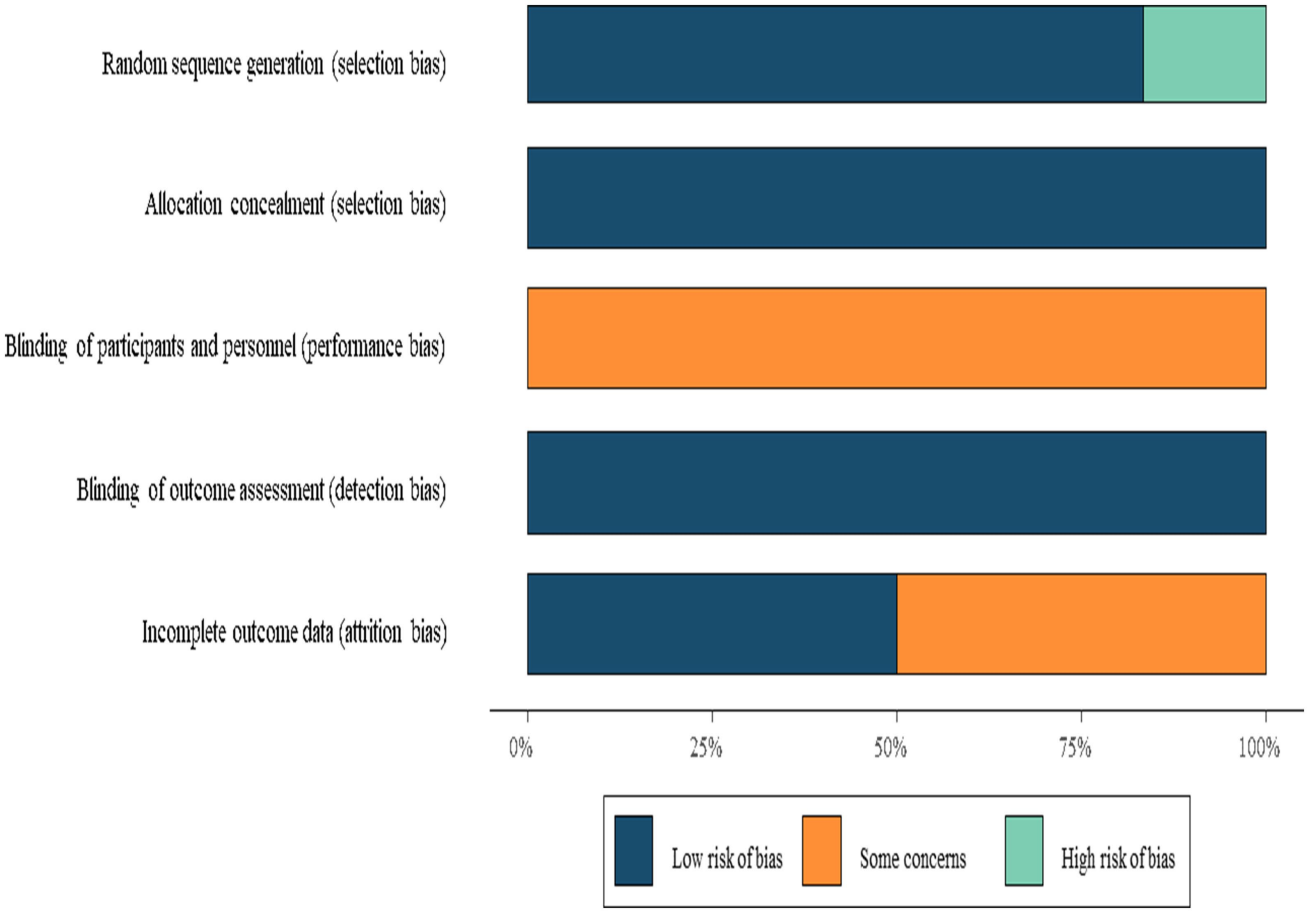
Risk of bias summary.

The six included studies comprised three two-arm trials and three three-arm trials. For multi-arm studies, only data pertinent to this meta-analysis were extracted. Interventions were categorized into three groups: conventional swallowing rehabilitation training (CT) (CT, e.g., effortful swallow, Mendelsohn maneuver, and skill-based training), swallowing training combined with neuromuscular electrical stimulation (NMES), and swallowing training combined with surface electromyographic biofeedback (sEMG-BF). For sEMG-BF, the specific exercise types varied across studies (see Table 2), including effortful swallow (16, 17), the Mendelsohn maneuver (18), and skill-based or task-specific swallowing exercises (17, 19–21). Methodological quality was assessed using the ROB tool. Due to the nature of the interventions, blinding of participants was not feasible, resulting in “some concerns” in this domain. All included studies were rated as moderate quality (Figure 2).

**Table 2:** Basic information of included literature.

Author (Ref)	Year	Enrollments	Intervention	Control	Sample Size	Type of swallowing exercise (with sEMG-BF)
Cheng (16)	2023	Dysphagia after acute hemorrhagic stroke	Swallowing training + sEMG-BF	Swallowing training + NMES	49 vs. 42	Effortful swallow
Gu (18)	2021	Post-stroke dysphagia	Swallowing training + sEMG-BF	① Swallowing rehabilitation training ② Swallowing training + NMES	40 vs. 40 vs. 40	Mendelsohn maneuver
Hou (19)	2024	Early post-stroke dysphagia	Swallowing training + sEMG-BF	Swallowing training + NMES	44 vs. 44	Skill-based swallowing training
Zhang (20)	2022	Post-stroke dysphagia	Swallowing training + sEMG-BF	Conventional swallowing training	32 vs. 32	Task-specific swallowing training
Min (17)	2014	Post-stroke dysphagia	Swallowing training + sEMG-BF	① Swallowing rehabilitation training ② Swallowing training + NMES	23 vs. 22 vs. 25	Effortful swallow
Wang (21)	2019	Post-stroke dysphagia	Swallowing training + sEMG-BF	① Swallowing rehabilitation training ② Swallowing training + NMES	20 vs. 20	Skill-based swallowing training

### 3.3 sEMG outcomes

**Figure 3:**
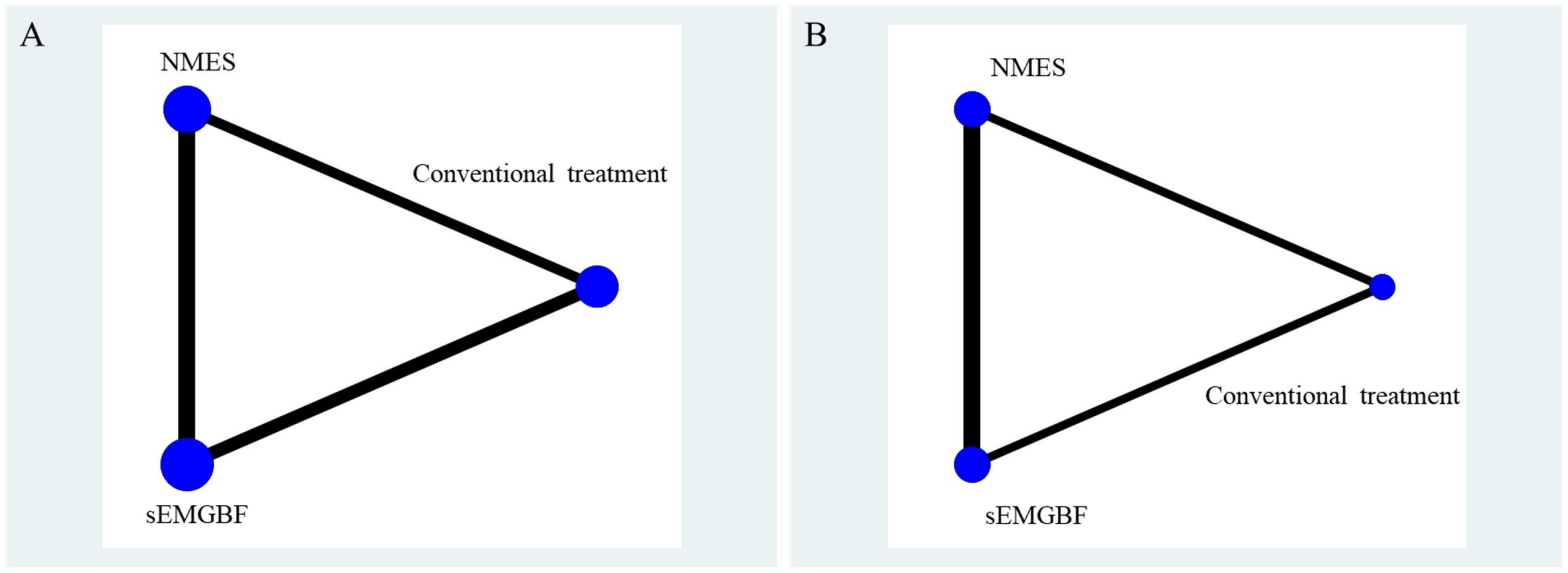
Network relationship diagram of surface electromyography results.

Post-intervention sEMG outcomes included mean amplitude (reported in six studies) and swallowing duration (reported in four studies). The network geometry (Figure 3) indicated that sEMG-BF was most frequently compared with NMES, followed by CT.

A represents average amplitude (μV); B represents swallowing duration(s).

**Figure 4:**
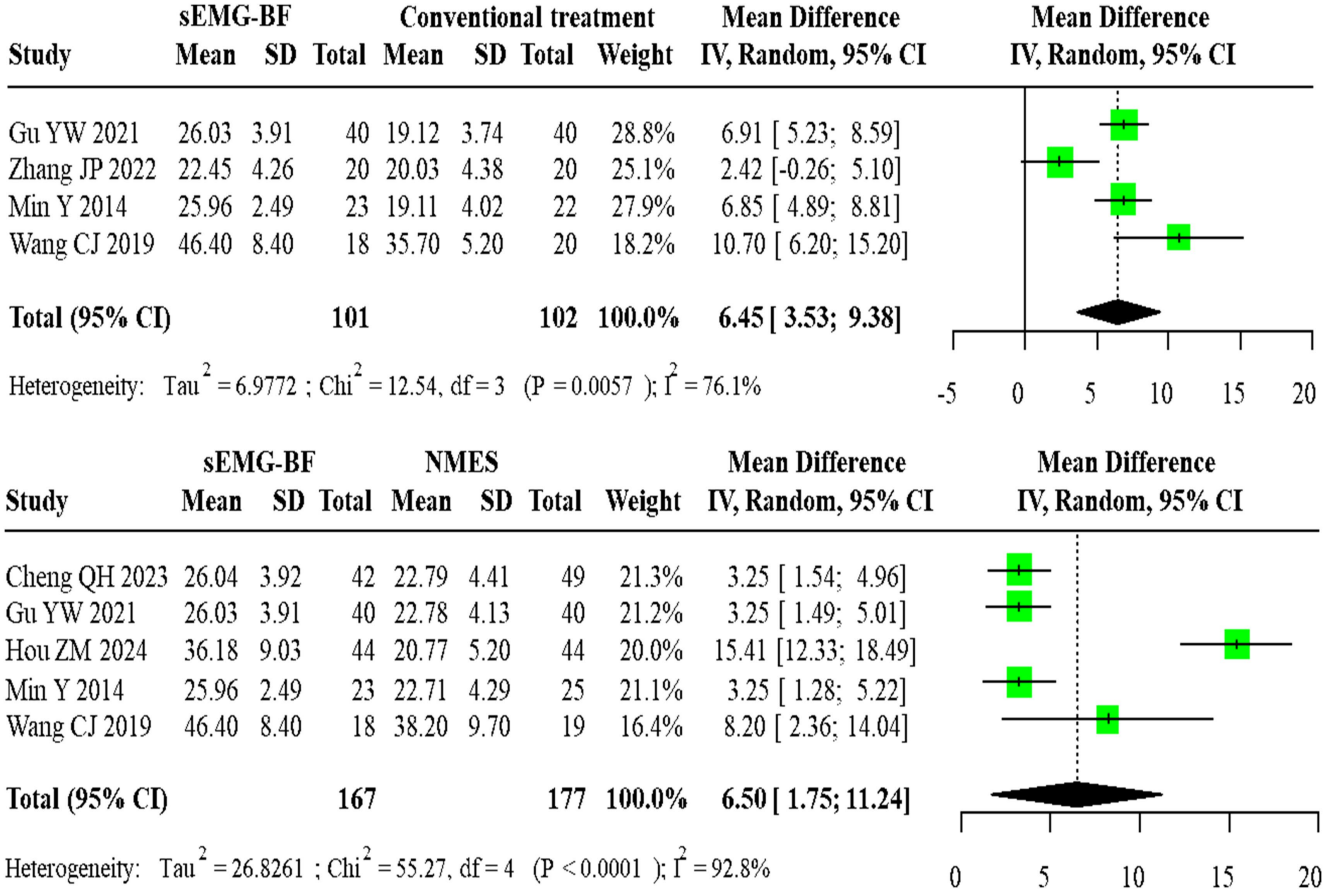
Forest plot of direct comparison meta-analysis for average amplitude.

For mean amplitude, four direct comparisons between sEMG-BF and CT showed significant heterogeneity (I^2^ > 50%), with a pooled MD of 6.45 (95% CI: 3.53–9.38) using a random-effects model. Five direct comparisons between sEMG-BF and NMES also exhibited heterogeneity, yielding an MD of 6.50 (95% CI: 1.75–11.24). Direct meta-analysis confirmed the superiority of sEMG-BF over both CT and NMES in improving mean amplitude (Figure 4).

**Figure 5:**
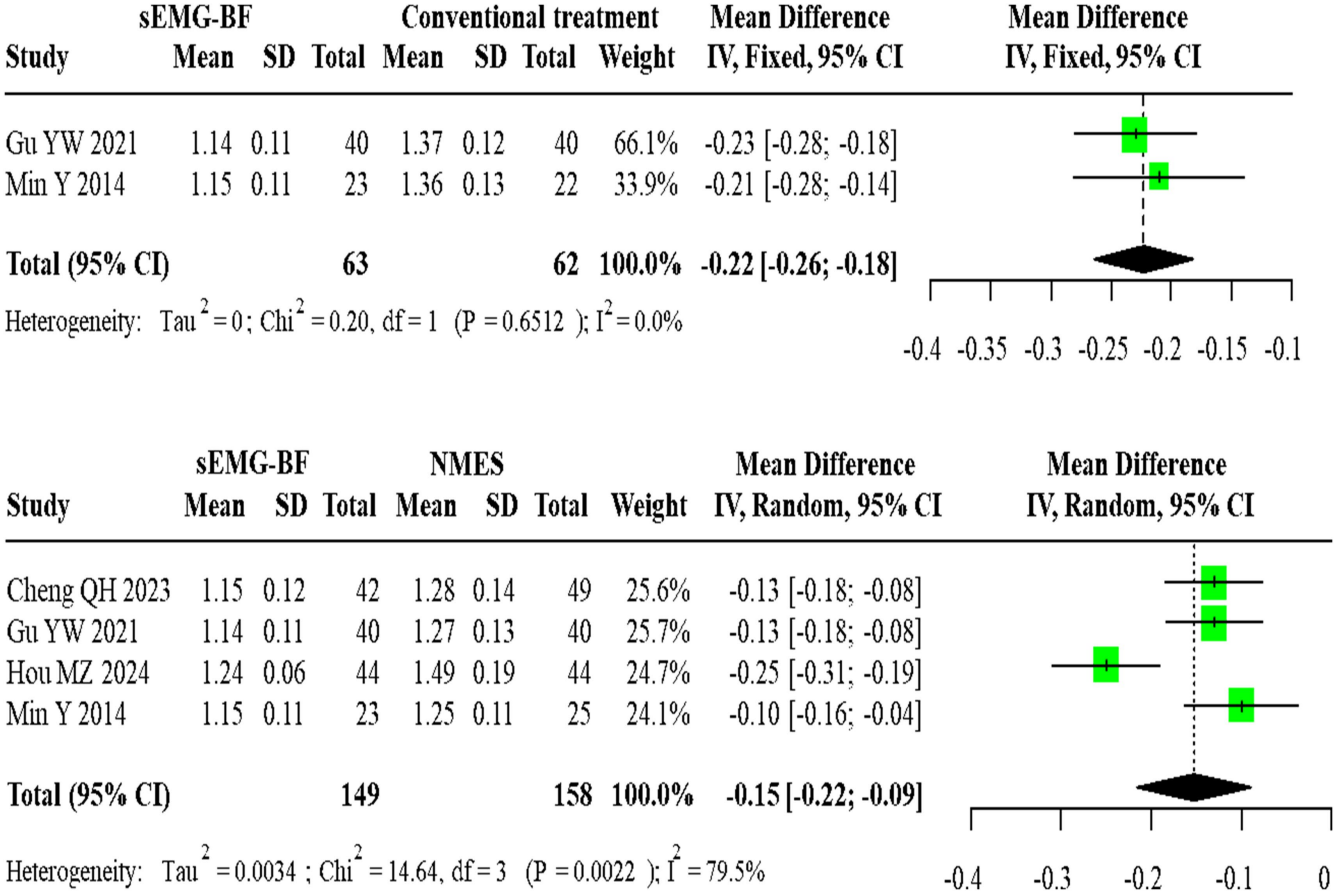
Forest plot of direct comparison meta-analysis for swallowing duration.

For swallowing duration, two homogeneous studies comparing sEMG-BF with CT (fixed-effect model) showed an MD of −0.22 (95% CI: −0.26 to −0.18). Four heterogeneous studies comparing sEMG-BF with NMES (random-effects model) demonstrated an MD of −0.15 (95% CI: −0.22 to −0.09). Direct meta-analysis revealed that sEMG-BF significantly reduced swallowing duration compared to both CT and NMES (Figure 5).

**Figure 6:**
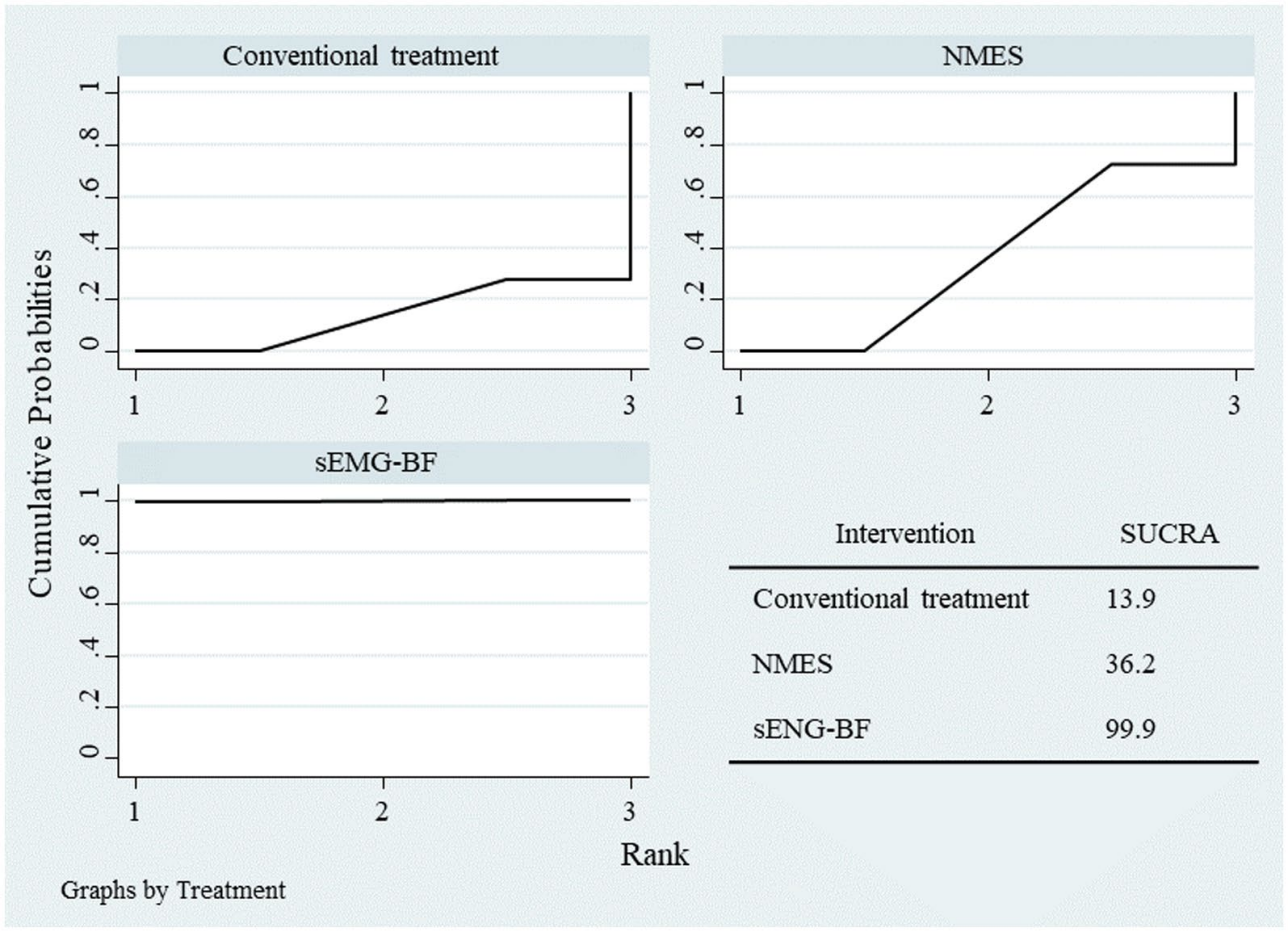
SUCRA plot for average amplitude.

**Figure 7:**
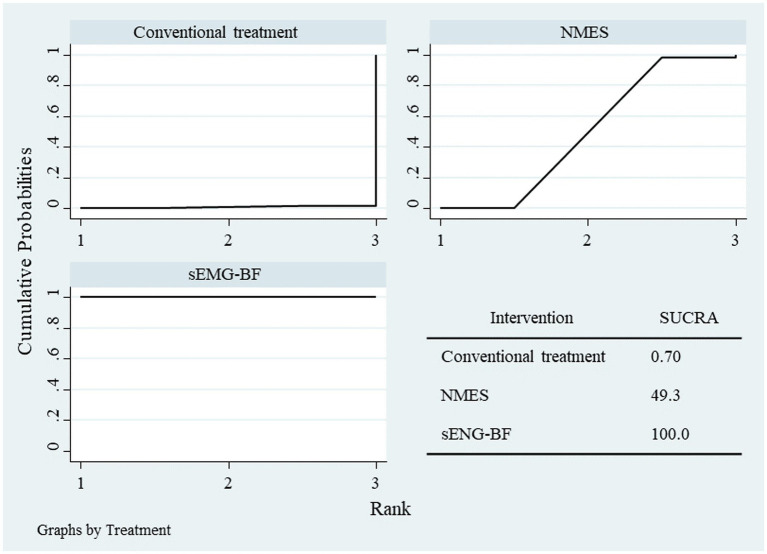
SUCRA plot for swallowing duration.

Network meta-analysis (NMA) results ranked the interventions as follows: sEMG-BF > NMES > CT for both mean amplitude (Figure 6) and swallowing duration (Figure 7). The analysis confirmed that sEMG-BF outperformed NMES and CT in improving amplitude and shortening swallowing duration, consistent with direct analysis findings. No significant differences were observed between NMES and CT for either outcome (Table 3).

**Table 3:** Results of network meta-analysis for amplitude and swallowing duration.

Parameters	Interventions	Mean difference	95%CI
Average amplitude	NMES vs. CT	1.36	−4.72, 7.67
	sEMGBF vs. CT	7.37	1.63, 13.25
	sEMGBF vs. NMES	5.96	0.91, 11.26
Swallowing duration	NMES vs. CT	−0.09	−0.21, 0.04
	sEMGBF vs. CT	−0.24	−0.36, −0.12
	sEMGBF vs. NMES	−0.15	−0.25, −0.06

### 3.4 Swallowing function and quality of life

**Figure 8:**
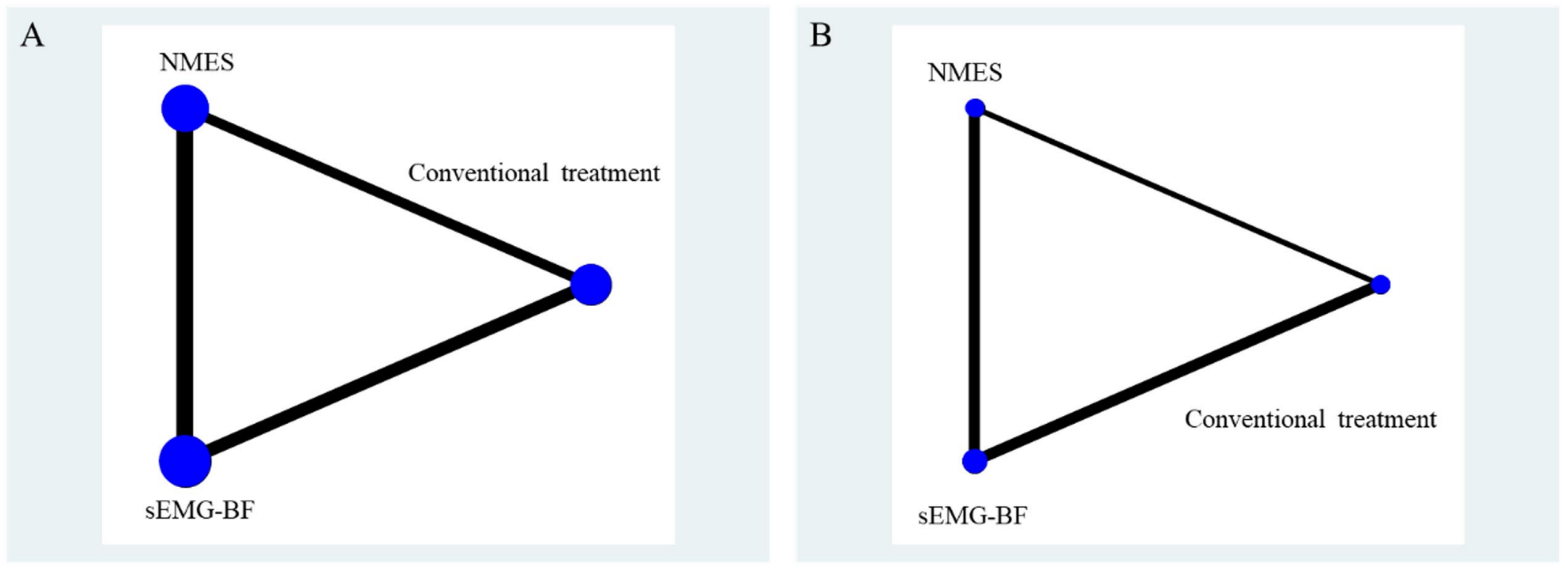
Network relationship diagram of swallowing function and quality of life results. **(A)** represents SSA score; **(B)** represents swallowing QOL scores.

Outcomes included the Standardized Swallowing Assessment (SSA) (five studies) and Swallowing Quality of Life (SWAL-QOL) (three studies) scores. The network graph is presented in Figure 8.

**Figure 9:**
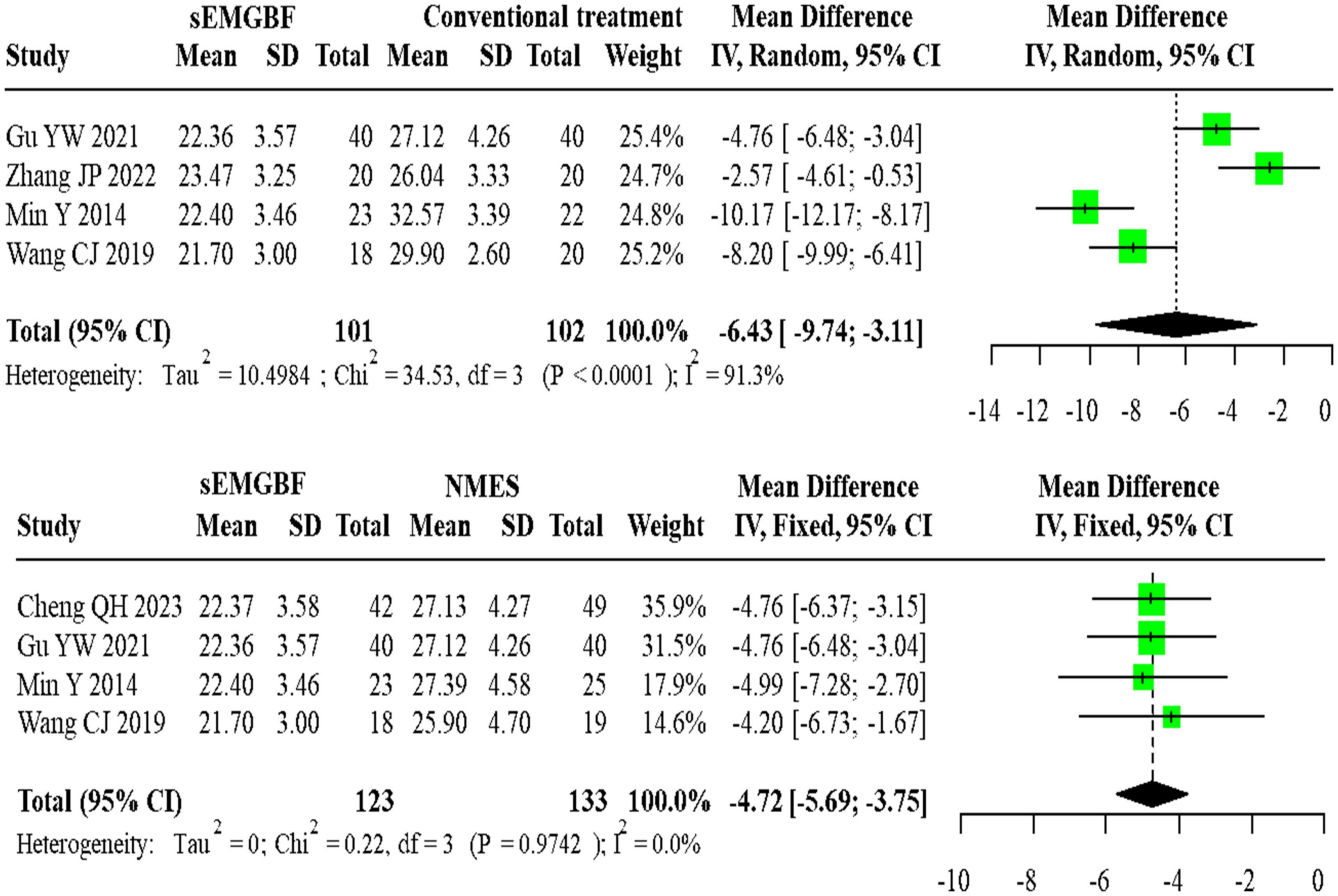
Forest plot of direct comparison meta-analysis for SSA scores.

For SSA scores, four heterogeneous studies comparing sEMG-BF with CT (random-effects model) showed an MD of −6.43 (95% CI: −9.74 to −3.11). Four homogeneous studies comparing sEMG-BF with NMES (fixed-effect model) yielded an MD of −4.72 (95% CI: −5.69 to −3.75). Direct meta-analysis indicated that sEMG-BF significantly improved swallowing function compared to both CT and NMES (Figure 9).

**Figure 10:**
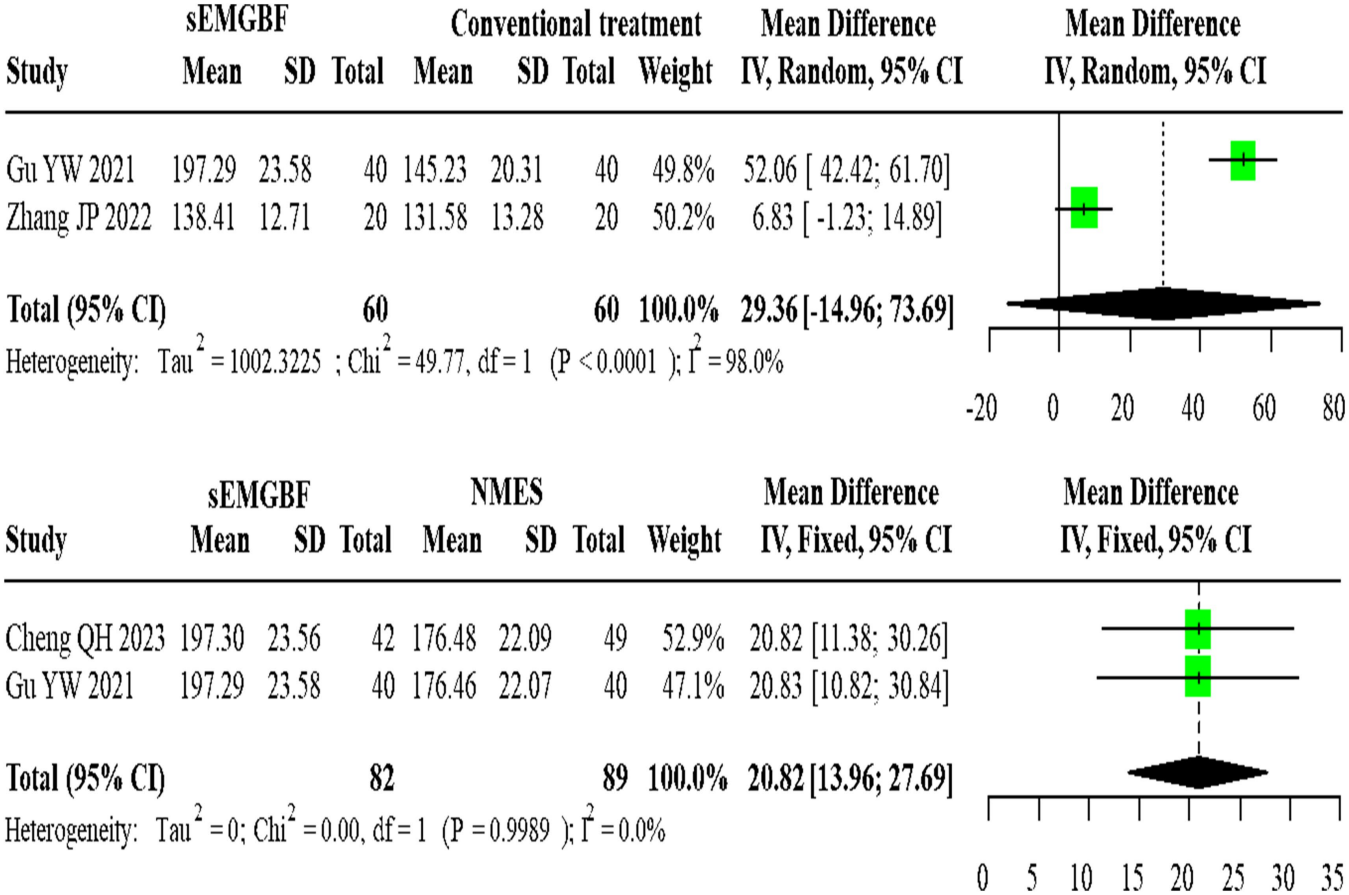
Forest plot of direct comparison meta-analysis for swallowing QOL scores.

For SWAL-QOL scores, two heterogeneous studies comparing sEMG-BF with CT (random-effects model) demonstrated an MD of 29.36 (95% CI: −14.96 to 73.69). Two homogeneous studies comparing sEMG-BF with NMES (fixed-effect model) showed an MD of 20.82 (95% CI: 13.96–27.69). Direct meta-analysis did not show a statistically significant difference in SWAL-QOL between sEMG-BF and CT or between sEMG-BF and NMES (Figure 10); for the sEMG-BF versus CT comparison, the pooled estimate (MD = 29.36, 95% CI − 14.96 to 73.69) crossed zero.

**Figure 11:**
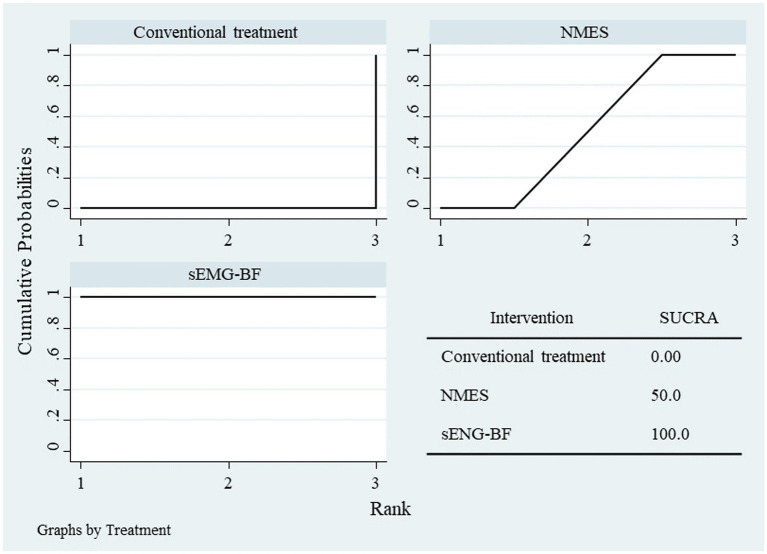
SUCRA plot for SSA scores.

**Figure 12:**
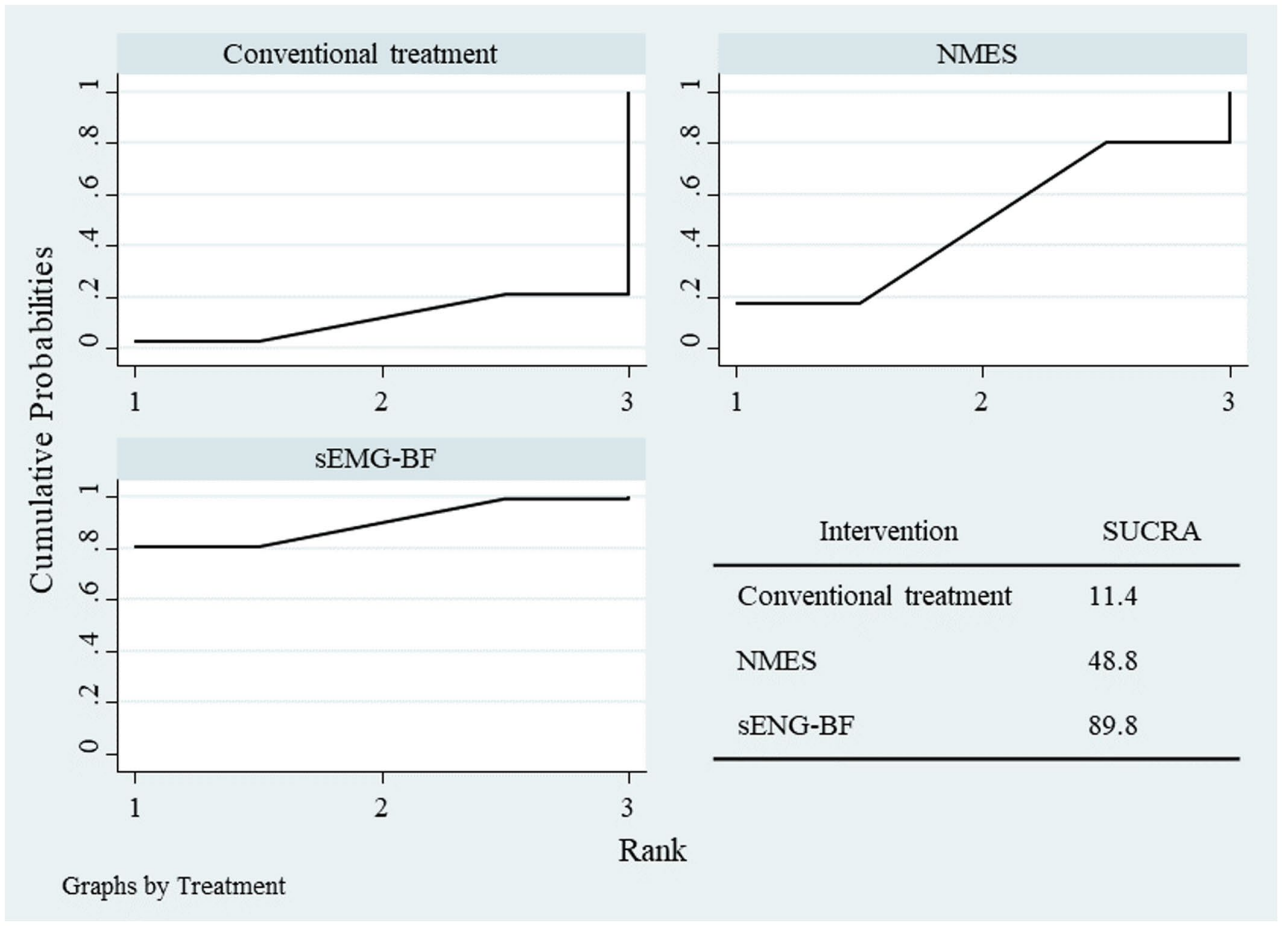
SUCRA plot for swallowing QOL scores.

NMA results ranked the interventions as sEMG-BF > NMES > CT for both SSA (Figure 11) and SWAL-QOL (Figure 12). The analysis confirmed that sEMG-BF was superior to NMES and CT in improving swallowing function, while NMES outperformed CT. No significant differences were observed among interventions for SWAL-QOL improvement (Table 4).

**Table 4:** Results of network meta-analysis for swallowing function and quality of life.

Parameters	Interventions	Mean difference	95%CI
SSA	NMES vs. CT	−3.87	−7.84, −0.15
	sEMGBF vs. CT	−7.90	−11.3, −4.14
	sEMGBF vs. NMES	−4.02	−7.55, −0.48
Swallowing QOL	NMES vs. CT	15.5	−40.8, 68.5
	sEMGBF vs. CT	30.6	−14.0, 78.3
	sEMGBF vs. NMES	15.3	−29.6, 61.4

## 4 Discussion

As an emerging rehabilitation technology, sEMG-BF has demonstrated considerable potential in rehabilitation, particularly in the recovery of neurological and motor functions (3). However, systematic evaluation and high-quality evidence are necessary to fully determine its efficacy and optimize its clinical application. This study aimed to conduct a systematic review and network meta-analysis to thoroughly investigate the therapeutic effects of sEMG-BF in PSD, clarify its efficacy advantages, and provide high-quality evidence to support clinical practice. The results suggest that sEMG-BF was superior to conventional therapy and NMES in improving swallowing function and sEMG parameters; however, the overall quality-of-life effects were not statistically significant, and the estimates were limited due to small sample sizes and heterogeneity. Specifically, sEMG-BF significantly increased the mean amplitude and reduced swallowing duration in sEMG metrics. Regarding swallowing function, sEMG-BF markedly improved the SSA score. In terms of quality of life, some studies suggested improvement with sEMG-BF; however, the pooled estimate did not achieve statistical significance, indicating uncertainty about this outcome. These findings indicate that sEMG-BF offers substantial clinical advantages in the treatment of PSD.

Previous studies have shown that traditional swallowing exercises, such as the Shaker exercise and Mendelsohn maneuver, although widely adopted, exhibit significant interindividual variability in efficacy due to limitations such as cognitive impairment or impaired proprioception in patients (8, 22–24). NMES can enhance muscle contraction through electrical stimulation but may cause discomfort or skin irritation and has minimal effects on the central nervous system (25). In the included studies, NMES was used as an independent intervention without biofeedback, in contrast to the sEMG-BF intervention. We recommend retaining these studies as comparators because NMES is a widely used clinical intervention and provides necessary context for evaluating the relative effectiveness of sEMG-BF. To avoid confusion, we have clarified in the discussion that NMES was applied without biofeedback. The findings of this study are consistent with prior research, further confirming the superiority of sEMG-BF in improving swallowing function and quality of life. sEMG-BF has been shown to significantly enhance swallowing function. A preliminary randomized controlled trial reported that patients receiving sEMG-BF rehabilitation exhibited improved pharyngeal transit efficiency and bolus clearance while simultaneously enhancing airway protection mechanisms during deglutition (11). Another study demonstrated that sEMG-BF combined with game-based training yielded greater improvements in swallowing indices compared to conventional rehabilitation alone (26). sEMG-BF facilitates increased muscle activity during swallowing exercises. One study found that both healthy participants and PSD patients generated significantly greater muscle activity when using sEMG biofeedback compared to non-biofeedback conditions, which is critical for effective swallowing rehabilitation (27). Moreover, sEMG-BF is generally well-accepted by patients, with high compliance. Studies indicate that the intervention is comfortable, manageable, and perceived as beneficial, making it a highly acceptable therapeutic approach (28).

sEMG-BF demonstrates potential benefits in improving various aspects of swallowing function, particularly in dysphagia patients, which can be attributed to multiple mechanisms. sEMG-BF is associated with enhanced upper esophageal sphincter (UES) function. One study found that sEMG-BF significantly prolonged UES relaxation duration and increased pharyngeal pressure, both of which are essential for effective swallowing (29). sEMG-BF combined with conventional swallowing training has been shown to significantly improve maximum hyoid displacement, a parameter closely associated with epiglottic elevation during swallowing (30). sEMG-BF effectively improves pharyngeal clearance, reduces residue, and enhances swallowing safety, which is particularly important in PSD patients (11). In addition, sEMG-BF provides real-time visual feedback, enabling patients to more accurately perceive and adjust swallowing muscle activity, thereby enhancing therapeutic efficacy. This approach overcomes the limitations of traditional swallowing exercises by offering a more intuitive and active training modality (31, 32). As a non-invasive technique, sEMG-BF has potential complementary utility with videofluoroscopic swallowing studies (VFSS) and may serve as an adjunctive tool for screening, diagnosing, and monitoring dysphagia. In patients with chronic low back pain, sEMG-BF can reduce muscle tension and improve physical function, thereby alleviating pain and enhancing sleep and mental health (33). Among patients suffering from spinal cord injuries, sEMG-BF can augment muscle activation and engagement (34).

Despite the comprehensive inclusion and analysis of relevant studies through systematic review and network meta-analysis, this study has several limitations. First, publication bias or the omission of unpublished negative results may affect the comprehensiveness of the findings. Second, the included studies exhibited considerable heterogeneity in patient characteristics, intervention details, and follow-up durations, which may influence the stability of the results. For instance, some studies had small sample sizes or short follow-up periods, limiting the interpretation of long-term effects. Furthermore, data on certain outcome measures, such as SWAL-QOL scores, were limited, potentially reducing the statistical power of the analysis.

Future research should focus on large-scale, long-term follow-up studies to evaluate the sustained efficacy and safety of sEMG-BF. In addition, the effectiveness of sEMG-BF may be influenced by the design and usability of electrodes and feedback systems, necessitating tailored approaches to maximize its benefits (35). Further exploration of combined therapies integrating sEMG-BF with other treatment modalities is warranted to optimize rehabilitation strategies for PSD. Feasibility studies on sEMG-BF in low- and middle-income countries are also recommended to assess its potential applicability in resource-limited settings.

## 5 Conclusion

This study indicates that sEMG-BF may be beneficial in the management of PSD. However, due to small sample sizes, heterogeneity, and limited data for some outcomes (e.g., SWAL-QOL), the evidence remains preliminary. Larger, high-quality studies are needed to confirm these findings. By enhancing the amplitude of sEMG signals and strengthening muscle contraction, sEMG-BF improves swallowing function; quality-of-life findings were inconclusive at the pooled level. Despite certain limitations, sEMG-BF holds promising prospects for the rehabilitation of PSD. Future high-quality studies are needed to further validate its long-term efficacy and safety and to explore its synergistic effects with other therapies to refine rehabilitation strategies.

## Data Availability

The raw data supporting the conclusions of this article will be made available by the authors, without undue reservation.
